# Quality of life outcomes in incidental and operated meningiomas (QUALMS): a cross-sectional cohort study

**DOI:** 10.1007/s11060-022-04198-y

**Published:** 2022-12-16

**Authors:** Sumirat M. Keshwara, Conor S. Gillespie, Mohammad A. Mustafa, Alan M. George, George E. Richardson, Abigail L. Clynch, Justin Z. Wang, David D. A. Lawson, Catherine E. Gilkes, J. Osman Farah, Jawad Yousaf, Emmanuel Chavredakis, Samantha J. Mills, Andrew R. Brodbelt, Gelareh Zadeh, Christopher P. Millward, Abdurrahman I. Islim, Michael D. Jenkinson

**Affiliations:** 1 Institute of Systems, Molecular & Integrative Biology, University of Liverpool, Liverpool, L69 7BE UK; 2 Department of Neurosurgery, The Walton Centre NHS Foundation Trust, Liverpool, L9 7LJ UK; 3 Division of Neurosurgery, Department of Surgery, University of Toronto, Toronto, ON Canada; 4 Department of Neuroradiology, The Walton Centre NHS Foundation Trust, Liverpool, L9 7LJ UK

**Keywords:** Meningioma, Active monitoring, Surgery, Quality of life

## Abstract

**Introduction:**

Few studies have evaluated meningioma patients’ longer-term health-related quality of life (HRQoL) following diagnosis and treatment, particularly in those with incidental, actively monitored tumours.

**Methods:**

A single-center, cross-sectional study was completed. Adult patients with surgically managed or actively monitored meningioma with more than five years of follow-up were included. The patient-reported outcome measures RAND SF-36, EORTC QLQ-C30 and QLQ-BN20 were used to evaluate HRQoL. HRQoL scores were compared to normative population data. Outcome determinants were evaluated using multivariate linear regression analysis.

**Results:**

243 patient responses were analyzed, and the mean time from diagnosis was 9.8 years (range 5.0–40.3 years). Clinically relevant, statistically significant HRQoL impairments were identified across several SF-36 and QLQ-C30 domains. Increasing education level (β = 2.9, 95% CI 0.9 to 4.9), *P =* .004), employment (β = 7.7, 95% CI 2.2 to 13.1, *P* = .006) and absence of postoperative complications (β=-6.7, 95% CI -13.2 to (-)0.3, *P* = .041) were associated with a better QLQ-C30 summary score. Other tumour and treatment variables were not.

**Conclusion:**

This study highlights the longer-term disease burden of patients with meningioma nearly one decade after diagnosis or surgery. Patients with actively monitored meningioma have similar HRQoL to operated meningioma patients. Healthcare professionals should be mindful of HRQoL impairments and direct patients to sources of support as needed.

**Supplementary Information:**

The online version contains supplementary material available at 10.1007/s11060-022-04198-y.

## Introduction

Meningioma is the most common primary intracranial tumour [1]. They are more common in women, and the incidence increases with age [1]. Patients with symptomatic or growing meningioma, or those that threaten neurovascular structures, are often managed with surgery. Patients with asymptomatic meningioma are usually monitored for growth using neuroimaging or symptom development [2].

The care for meningioma patients has steadily improved over the past three decades due to a better understanding of the natural history and disease biology, advances in surgical frameworks and increasing adjuvant and salvage therapy options [3–5]. The overall 10-year age-adjusted UK survival rates for WHO Grade 1, 2 and 3 meningiomas are 81%, 63% and 15%, respectively [6]. Traditionally, clinical outcomes for meningioma patients have been reported in terms of mortality, morbidity, and disability. However, these methods of clinical outcome assessment are by themselves insufficient, and modern clinical outcome assessment is increasingly patient-centred [7].

Patient-reported outcomes (PROs) are outcomes reported directly by a patient about the status of their health condition or perception of a disease and its treatment [8]. Health-related quality of life (HRQoL) is a PRO that assesses how a patient’s physical, emotional and social well-being are affected by a medical condition or treatment [9]. HRQoL is a multidimensional construct that can be measured by assessing a range of patient-reported functioning and well-being domains [10].

Multiple studies have reported perioperative and short-term postoperative HRQoL outcomes in meningioma patients [11–22]. Overall, these studies show that patients have worse HRQoL compared to controls or normative patient values. However, few studies have evaluated the longer-term (≥ 5 years) HRQoL of surgically treated meningioma patients [23–25]. No studies have specifically evaluated the longer-term HRQoL of patients under active monitoring for incidental meningioma.

## Objective

The primary objective of this study was to evaluate longer-term HRQoL and its determinants in actively monitored and operated meningioma patients.

## Methods

### Study design, setting and participants

This was a single-centre, cross-sectional study (The QUALMS study) conducted at The Walton Centre NHS Foundation Trust, Liverpool, United Kingdom. Two patient cohorts were included: those with actively monitored meningioma and those with surgically operated meningioma. General inclusion criteria were age ≥ 16 years at the time of diagnosis and the ability to communicate effectively in English. Patients in the actively monitored cohort were included if they had a radiological diagnosis of meningioma with a minimum follow-up of 5 years without intervention. Patients in the operated cohort were included if they had surgery for meningioma and a minimum of 5 years subsequent follow-up, with no limit on additional disease control or interventions received. Patients lacking the capacity to consent to study participation, or those with a neurological disease or insult before meningioma diagnosis, radiation-induced meningioma, neurofibromatosis type-2 associated meningioma or any other condition leading to cognitive decline were excluded. The study was conducted according to the guidelines of the Declaration of Helsinki and approved by the Research Ethics Committee of the National Health Service (NHS) Health Research Authority (HRA) (REC: 19/SW/0181, IRAS: 269742) on 16 October 2019.

### Data collection

Assessment of HRQoL five or more years after diagnosis or surgery was chosen as the time point for obtaining longer-term HRQoL outcomes. Patients were approached in one of two ways: either when they attended routine clinic appointments or by post. Patient consent was obtained before obtaining questionnaire responses, either in clinic or by post. Following completion of consent forms, patients were given study HRQoL questionnaires. The study HRQoL questionnaires were the RAND 36-Item Short Form Health Survey (SF-36) 1.0, EORTC Core Quality of Life of Cancer Patients Questionnaire (EORTC QLQ-C30) 3.0 and brain tumor module (EORTC QLQ-BN20). These questionnaires were completed by patients to evaluate HRQoL. Additionally, a study-specific questionnaire was completed by patients to collect the following data: education level, employment status, driving license status, driving status, currently under follow-up for meningioma, last clinic appointment date, medical conditions, and regular medications. Patients completed questionnaires between December 2019 and March 2021. A retrospective chart review collected patients’ demographic, tumour, and treatment variables.

### HRQoL questionnaires

The RAND SF-36 1.0 was chosen as it is a validated, generic HRQoL assessment tool [26, 27, 47]. The RAND SF-36 contains 36 items and assesses eight HRQoL domains: physical functioning, role limitations due to physical health, role limitations due to emotional problems, energy/fatigue, emotional well-being, social functioning, pain and general health. A higher domain score represents a more favourable health state.

The EORTC QLQ-C30 3.0 was chosen as it is a validated, cancer-specific HRQoL assessment tool [28]. The QLQ-C30 assess five functional scales, three symptom scales and one global health status/ QoL scale. Six single items evaluate the burden from common cancer symptoms. A higher score on functional scales and global health status indicates a more favourable health state, whereas a higher symptom scale or single item score indicates a higher symptom burden. It is possible to compute a QLQ-C30 summary score by combining the scores of the following scales and single items: physical functioning, role functioning, social functioning, emotional functioning, cognitive functioning, fatigue, pain, nausea and vomiting, dyspnoea, insomnia, appetite loss, constipation and diarrhoea. A higher QLQ-C30 summary score represents a higher HRQoL.

The EORTC QLQ-BN20 is an extension of the QLQ-C30 and was chosen as it is a validated, brain tumour-specific HRQoL assessment tool [29, 30]. The QLQ-BN20 consists of four symptom scales (future uncertainty, visual disorder, motor dysfunction, communication deficit) and seven single items (headache, seizure, drowsiness, hair loss, itchy skin, leg weakness, bladder control) which evaluate the burden from common brain cancer symptoms. A higher score represents a higher level of symptom burden.

To allow standardization of raw scores for statistical analysis, raw scores from each HRQoL domain or item were transformed to produce scores on a scale ranging from 0 to 100.

### Missing data

Where there were missing items within a RAND SF-36 domain, the remaining answered items were averaged to compute the domain score. Therefore, no patients were excluded from the analysis of RAND SF-36 domains. Where greater than 50% of items within a scale were missing from EORTC QLQ-C30 or QLQ-BN20, patients were excluded from analyses of that scale. Therefore, one patient was excluded from analyses of the EORTC QLQ-C30 physical functioning scale and one patient from analyses of EORTC QLQ-BN20 visual disorder. To calculate an EORTC QLQ-C30 summary score, a patient must have a valid score within the included scales and items. Twelve patients were removed from the EORTC QLQ-C30 summary score analysis as they lacked valid scores in at least one of the scales or items required to compute the score.

### Statistical analysis

Statistical analysis was completed using IBM SPSS Statistics (Version 26.0, Released 2019, IBM Corp., Armonk, NY). Using the primary outcome of the study ‘to identify longer-term HRQoL outcomes in actively monitored or operated meningioma patients compared to normative population values’, the sample size was estimated as 170 meningioma patients to identify a minimal clinically important difference (MCID) of 5 points between HRQoL domains of patients and the normative population, assuming type 1 error = 5% and type 2 error = 10%. This assumes the mean HRQoL domain scores of the general population are 50 and the standard deviation is 10. Visual inspection of histograms and the Shapiro-Wilk test were used to assess for skewness and normality, respectively. Comparison between meningioma patients and unadjusted normative population values were completed using Student’s t-test. Normative population values for SF-36 and EORTC QLQ-C30 were obtained from published literature. [31, 32] No normative population data was available for the QLQ-BN20, so the results of patients from this study are presented without comparison to any other population. As MCIDs for the chosen questionnaires in the meningioma population are unknown, we set the MCIDs at 5 points.

Backward stepwise multivariate linear regression was used to identify variables associated with impaired HRQoL domains. For the model, each variable was first analyzed using univariate regression analysis and incorporated into the multivariate model if the *P*-value was less than 0.10. The following variables were evaluated in regression analysis: sex, Age-adjusted Charlson Comorbidity Index (ACCI) at diagnosis, WHO Performance Status, education level, employment, response during or after UK COVID-19 lockdown (response on or after 23 March 2020), incidentally diagnosed meningioma, skull base tumour, tumour laterality, presence of multiple meningiomas, number of antiepileptic drugs (AEDs), intervention received for meningioma, number of surgeries, postoperative complications, number of radiotherapy courses (fractionated radiotherapy [FRT] or stereotactic radiosurgery [SRS]), type of radiotherapy (FRT, SRS or both) and duration of follow-up. WHO Grade was not included in the multivariate analysis as not all meningioma patients had WHO Grade data available (e.g., actively monitored patients). EORTC QLQ-C30 summary score was chosen as the dependent variable as it is a composite score comprised of several HRQoL scales and items. A *post hoc* univariate and multivariate linear regression analysis was completed separately for the surgical and non-surgical patient cohorts. No correction for multiple testing was performed in this explorative study to reduce the risk of a type 2 error. [33] A *P*-value of less than 0.05 was considered statistically significant.

## Results

### Patient population

Patient demographic data are presented in Table 1. Six hundred and ninety-nine patients were invited to participate by post within the study period, and additional patients were approached at routine clinic appointments. Two hundred and forty-seven responded, and 243 had baseline demographic data available permitting analysis. The median age of participants was 69.2 years (IQR = 58.4–74.8, range: 31.5–93.6). Most participants were female (n = 195, 80.2%). The mean time from diagnosis to completion of questionnaires was 9.8 years (SD 22.2, range: 5.0–40.3). Incidentally diagnosed meningioma patients constituted approximately half of the cohort (n = 112, 46.1%). Most participants were WHO Performance Status 0 (n = 110, 45.3%) and 1 (n = 95, 39.1%). At the time of questionnaire completion, most participants were retired (n = 143, 58.8%) or in employment (n = 29, 11.9%), though some were unable to work due to health reasons (n = 23, 9.5%). Most patients were under active follow-up for their meningioma (n = 144, 59.3%).

**Table 1:** Demographic data of study respondents

Baseline characteristics	Value
Total patients	243
Multiple meningioma (%)	18 (7.4)
Female (%)	195 (80.2)
Median age at completion (IQR)	69.2 (58.4–74.8)
Years of follow up (SD)	9.8 (22.2)
Incidentally discovered meningioma (%)	112 (46.1)
**WHO Performance Status**	**Frequency (%)**
0	110 (45.3)
1	95 (39.1)
2	30 (12.3)
3	4 (1.6)
**Age-adjusted Charlson Comorbidity Index at diagnosis**	**Frequency (%)**
0–2	154 (63.4)
3–5	75 (30.9)
6+	14 (5.8)
**Employment status**	**Frequency (%)**
Full-time employment	29 (11.9)
Part-time employment	21 (8.6)
Self-employed	11 (4.5)
Homemaker	9 (3.7)
Retired	143 (58.8)
Unable to work due to health	23 (9.5)
Not working (other)	4 (1.6)
**Education**	**Frequency (%)**
No education	38 (15.6)
School	92 (37.9)
College	51 (21.0)
University	24 (9.9)
Postgraduate qualification	31 (12.8)
**Recruitment period**	**Frequency (%)**
Before UK COVID-19 lockdown	27 (11.1)
During & after UK COVID-19 lockdown	212 (87.2)
**WHO Grade**	**Frequency (%)**
1	125 (85.0)
2	22 (15.0)
**Radiological variables**	**Frequency (%)**
Left-sided	105 (43.2)
Right-sided	94 (38.7)
Midline	34 (14.0)
Skull base	93 (38.3)
**Interventions**	**Frequency (%)**
Any treatment	159 (65.4)
Surgery	156 (64.2)
Radiotherapy	41 (16.9)
Anti-epileptic drug use	31 (12.8)

**Fig. 1:**
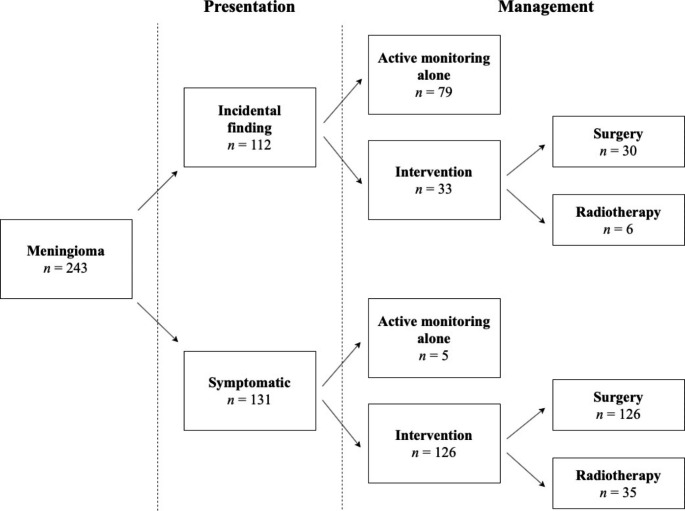
A diagram summarizing the management of patients stratified by presentation

During the follow-up period, 156 patients (64.2%) had at least one operation for their meningioma. Of the patients who had at least one operation, most had initially presented with symptoms (n = 126, 80.8%). Radiotherapy was given to 41 patients (16.9%). Of those undergoing surgery, 147 had WHO Grade data available: 125 patients (85.0%) had WHO Grade 1 meningioma, and 22 patients (15.0%) had WHO Grade 2. Of patients with incidentally diagnosed meningioma, 79 patients (70.5%) were managed with active monitoring alone. Figure 1 illustrates the number of patients receiving either intervention or active monitoring alone for their meningioma, stratified by their presentation.

### HRQoL of meningioma patients

HRQoL results of meningioma patients are presented in Table 2. Supplementary Table 1 reports the skewness of HRQoL data and results of normality testing. QLQ-C30 symptom scores were generally positively skewed, and QLQ-C30 functional scale and SF-36 scores were negatively skewed. HRQoL scores were not normally distributed as assessed by with Shapiro-Wilk test (*P* values < 0.05).

**Table 2:** Respondent HRQoL scores compared to normative population data (SF-36 and EORTC QLQ-C30). EORTC QLQ-BN20 results are presented without comparison

Scale/ item	Number of responses	Cohort score (SD)	Normative score (SD)	*P* value	Mean difference
**SF-36**					
Physical Functioning	239	64.92 (31.94)	66.2 (31.4)	0.582	-1.28
Emotional Well-being	239	71.41 (20.35)	76.2 (18.1)	< **0.001** *	-4.79
Social Functioning	237	70.99 (32.71)	77.5 (22.7)	**0.004** *	-6.51 **
Role Physical	232	57.76 (44.00)	67.7 (34.7)	**0.002** *	-9.94 **
Role Emotional	233	69.89 (41.24)	84.0 (28.4)	< **0.001** *	-14.11 **
Energy/ Fatigue	240	52.03 (25.09)	57.7 (22.7)	**0.002** *	-5.67 **
Pain	237	63.80 (29.28)	62.0 (28.9)	0.395	1.80
General Health	241	56.47 (24.86)	61.3 (24.2)	**0.007** *	-4.83
**EORTC QLQ-C30**					
Physical Functioning	238	76.43 (25.23)	81.8 (23.5)	**0.003** *	-5.37 **
Emotional Functioning	237	72.22 (26.77)	71.0 (28.4)	0.532	1.22
Social Functioning	237	77.14 (32.17)	80.3 (29.4)	0.167	-3.16
Cognitive Functioning	236	71.12 (29.26)	80.5 (25.2)	< **0.001** *	-9.38 **
Role Functioning	238	74.58 (32.57)	80.2 (29.1)	**0.015** *	-5.62 **
Fatigue	239	35.05 (28.55)	32.2 (27.6)	0.163	2.85
Nausea & Vomiting	238	6.37 (15.24)	8.1 (18.9)	0.133	-1.73
Pain	239	33.82 (32.68)	26.7 (31.2)	**0.002** *	7.12 **
Dyspnoea	237	18.00 (28.03)	19.5 (27.9)	0.458	-1.50
Insomnia	236	35.88 (36.30)	32.6 (32.8)	0.205	3.28
Appetite Loss	238	12.47 (25.22)	14.2 (25.2)	0.341	-1.73
Constipation	239	12.97 (22.95)	14.7 (26.2)	0.308	-1.73
Diarrhoea	237	7.74 (18.44)	11.2 (23.0)	**0.014** *	-3.46
Financial Difficulties	235	8.09 (21.03)	14.5 (28.7)	< **0.001** *	-6.41 **
Global Health	237	66.98 (23.21)	62.3 (23.7)	**0.006** *	4.68
Summary Score	231	77.82 (18.98)	-	-	-
**EORTC QLQ-BN20**					
Future Uncertainty	237	25.80 (24.42)	-	-	-
Visual Disorder	237	13.71 (21.14)	-	-	-
Motor Dysfunction	237	17.39 (21.99)	-	-	-
Communication Deficit	237	14.72 (20.17)	-	-	-
Headache	237	25.88 (31.53)	-	-	-
Seizure	236	1.84 (11.15)	-	-	-
Drowsiness	235	27.94 (29.55)	-	-	-
Hair Loss	234	11.54 (25.37)	-	-	-
Itchy Skin	235	14.18 (24.21)	-	-	-
Leg Weakness	236	14.97 (26.84)	-	-	-
Bladder Control	236	17.51 (29.72)	-	-	-

* Indicates a significant result (also underlined and in bold). ** Indicates a clinically significant result.

Compared to the SF-36 normative population data, meningioma patients reported clinically relevant, statistically significant, worse HRQoL scores in four RAND SF-36 domains: role limitations due to physical health problems (mean difference = -9.94, *P* = .002), role limitations due to emotional health problems (mean difference = -14.11, *P* < .001), energy/fatigue (mean difference = -5.67, p = .002) and social functioning (mean difference = – 6.51, *P* = .004). Statistically significant, but not clinically relevant impairments were identified for meningioma patients in the following domains: emotional well-being (mean difference = -4.79, *P* < .001) and general health (mean difference = -4.83, *P* = .007).

Compared to EORTC QLQ-C30 normative population data, meningioma patients reported clinically relevant, statistically significant, impaired HRQoL scores in four domains: physical functioning (mean difference = -5.37, *P* = .003), role functioning (mean difference = -5.62, *P* = .015), cognitive functioning (mean difference = -9.38, *P* < .001) and pain (mean difference = 7.12, *P* = .002). Patients reported clinically relevant and statistically significant fewer financial difficulties than the normative population (mean difference = – 6.41, *P* < .001). Patients reported statistically significant, but not clinically relevant, better diarrhoea (mean difference = -3.46, *P* = .014) and global health scores (mean difference = 4.68, *P* = .006).

### Variables associated with HRQoL of meningioma patients

The results of the multivariate linear regression analysis to identify variables associated with EORTC QLQ-C30 summary score are presented in Table 3. Three variables were significantly associated with better QLQ-C30 summary scores: male sex: (β = 6.5, CI 0.5 to 12.4, *P* = .033), increasing education level (β = 2.9, 95% CI 0.9 to 4.9, *P* = .004), and patients in employment (β = 7.7, 95% CI 2.2 to 13.1, *P* = .006). The presence of postoperative complications was associated with a worse QLQ-C30 summary score (β = -6.7, 95% CI -13.2 to -0.3, *P* = .041). No other demographic, tumour or treatment variables were significantly associated with the QLQ-C30 Summary Score on multivariate analysis. When compared to patients that responded before the UK COVID-19 lockdown, those who responded during or after the lockdown did not have significantly different QLQ-C30 summary scores in univariate analysis (β = -2.3, 95% CI -10.0 to 5.4, *P* = .553). Duration of follow-up (defined as the time from diagnosis to completion of questionnaires) was not significantly associated with QLQ-C30 summary score in univariate analysis (β = 0.2, 95% CI -0.5 to 0.9, *P* = .586).

**Table 3:** Results of the univariate and multivariate linear regression analysis to identify variables significantly associated with EORTC QLQ-C30 summary score

	Univariate analysis		Multivariate analysis	
**Variable**	**Beta coefficient (95% CI)**	***P*** **value**	**Beta coefficient (95% CI)**	***P*** **value**
Male sex	6.7 (0.6 to 12.8)	**0.032** *	6.5 (0.5 to 12.4)	**0.033** *
ACCI at diagnosis	-1.4 (-2.8 to -0.1)	**0.041** *	0.2 (-1.5 to 1.9)	0.816 ^a^
Performance status	-3.7 (-7.0 to -0.5)	**0.026** *	-2.5 (-5.7 to 0.8)	0.142 ^b^
Education level	3.7 (1.8 to 5.7)	**< 0.001** *	2.9 (0.9 to 4.9)	**0.004** *
Employment	9.4 (3.9 to 14.9)	**0.001** *	7.7 (2.2 to 13.1)	**0.006** *
Response during/ after COVID-19 lockdown	-2.3 (-10.0 to 5.4)	0.553	-	-
Incidental meningioma	-1.7 (-6.6 to 3.3)	0.501	-	-
Skull base	0.221 (-4.9 to 5.4)	0.932	-	-
Tumour laterality	-0.544 (-4.2 to 3.1)	0.769	-	-
Multiple meningioma	3.5 (-5.9 to 13.0)	0.462	-	-
Number of AEDs	-2.4 (-8.0 to 3.3)	0.410	-	-
Intervention	1.8 (-3.4 to 7.0)	0.493	-	-
Number of surgeries	-0.4 (-4.6 to 3.8)	0.849	-	-
Postoperative complications	-7.4 (-14.0 to -0.8)	**0.027** *	-6.7 (-13.2 to -0.3)	**0.041** *
Number of radiotherapy courses	-1.2 (-6.9 to 4.5)	0.671	-	-
Type of radiotherapy	-1.5 (-4.9 to 2.0)	0.401	-	-
Duration of follow up	0.2 (-0.5 to 0.9)	0.586	-	-

* Indicates a significant result (also underlined and in bold). ^a^ Variable excluded at step 1 of multivariate backward linear regression analysis. ^b^ Variable excluded at step 2 of multivariate backward linear regression analysis.

### Variables associated with HRQoL in surgically and non-surgically managed meningioma patients

The results of the *post hoc* univariate and multivariate linear regression analysis to identify variables associated with EORTC QLQ-C30 summary score in surgical and non-surgical cohorts are presented in Supplementary Tables 2 and 3. For the surgical cohort, male sex (β = 8.8, 95% CI 2.0 to 15.7, *P* = .012), an increasing education level (β = 2.7, 95% CI 0.5 to 4.8, *P* = .014) and patients in employment (β = 6.5, 95% CI 0.1 to 12.9, *P* = .045) were significantly associated with better QLQ-C30 summary scores. The presence of postoperative complications was associated with a worse QLQ-C30 summary score (β =-7.9, 95% CI -14.4 to -1.4, *P* = .017). For the non-surgical cohort, only increasing education level was associated with better QLQ-C30 summary scores (β = 4.5, 95% CI 0.2 to 8.7, *P* = .040). No variables were significantly associated with worse QLQ-C30 summary scores in the non-surgical cohort.

## Discussion

Within the neuro-oncology literature, several studies have evaluated long-term HRQoL of different CNS tumours. One recent study of low-grade glioma showed that patients reported similar or better HRQoL scores when compared to controls two decades following diagnosis [34]. Another study of long-term HRQoL in vestibular schwannoma patients showed the diagnosis of the tumour rather than the treatment strategy most significantly impacts HRQoL [35]. However, few studies have investigated longer-term HRQoL in meningioma patients, particularly those undergoing an active monitoring strategy. This study was designed to evaluate the long-term HRQoL of actively monitored and surgically operated meningioma patients.

### Summary of findings

This study has identified inferior longer-term HRQoL outcomes of both actively monitored and operated meningioma patients nearly a decade after diagnosis or treatment. In many scales of the SF-36, QLQ-C30 and QLQ-BN20, these impairments were both clinically relevant and statistically significant. Increasing education level and employment were associated with better overall HRQoL scores, as assessed by the QLQ-C30 summary score, and the presence of postoperative complications was associated with worse overall HRQoL scores. Diagnosis of incidental meningioma and use of radiotherapy are two variables that were not associated with overall long-term HRQoL scores.

### Interpretation of findings

Several domains across the SF-36 and QLQ-C30 were impaired compared to normative population values. Many of these were clinically relevant. This supports the findings of another large study of operated meningioma patients, which also demonstrated long-term HRQoL sequelae [23]. Many studies have identified significant HRQoL impairments in the short and medium-term [21, 36, 37]. This study provides additional evidence of longer-term impairments in a cohort of both actively monitored and operated meningioma. Healthcare professionals should acknowledge that functioning and well-being issues can exist in patients at every disease phase and therefore attempt to identify possible HRQoL impairments during follow-up. Given that impairments of HRQoL have been identified up to a decade after diagnosis and treatment, future studies should focus on strategies to identify and improve functioning and well-being. Meningioma patients may benefit from regular holistic needs assessments to improve well-being, led by either specialist nurses or doctors, as part of their routine follow-up.

Diagnosis of an incidental meningioma or active monitoring management strategy was not associated with the EORTC QLQ-C30 summary score. In a study assessing medium-term HRQoL on average 2–3 years post-diagnosis, actively monitored meningioma patients reported inferior SF-36 general health and physical component scale aggregate scores compared to operated meningioma patients [38]. However, in a study of longer-term HRQoL, no difference was identified between scores of patients managed with active monitoring (n = 12) and those who had first-line surgery or radiotherapy [23]. This observation may be explained by improvement in HRQoL in actively monitored patients, or deterioration in HRQoL in the operated patients, over time. Future studies could prospectively elucidate the changes in HRQoL for both actively monitored and operated meningioma patients. Initially, after diagnosis and treatment, several limitations may affect one’s HRQoL. These limitations may be different between actively monitored and operated patients. Over time patients may re-evaluate what they consider normal (response-shift effect) and this may explain the equalisation of the HRQoL metrics between meningioma patients. Our findings support the hypothesis that in the longer-term, both actively monitored and operated meningioma patients experience similar levels of HRQoL burden. In a study of untreated meningiomas, of the patients without mental health disorders at the time of meningioma diagnosis, 16% went on to develop a mental health disorder within the first year following meningioma diagnosis [48]. Therefore, patients with incidental, actively monitored meningioma should receive the same level of screening as operated patients for well-being issues as part of their routine follow-up and directed toward support services as required.

Radiotherapy (FRT or SRS) was not associated significantly with the QLQ-C30 summary score. This is similar to three studies of longer-term HRQoL in meningioma patients showing that adjuvant fractionated radiotherapy was not associated with SF-36 physical or mental component scale summary scores [23, 25, 39]. By contrast, when assessing HRQoL scores in the early postoperative phase (six months), one study of 1722 patients identified lower HRQoL scores in the SF-36 domains of vitality, role physical and social functioning for patients who received radiotherapy compared to patients who had surgery only [21]. Additionally, in the perioperative period, patients undergoing stereotactic radiosurgery were found to have worse SF-36 HRQoL scores pre-treatment, which improved at 6 months, and then began to decrease to baseline values at 12 months [40]. Overall, the results of this study combined with the literature may suggest that patients have lower HRQoL following radiotherapy in the short-term but not in the longer-term when compared to those who have not received radiotherapy.

The multivariate analysis showed that patients in employment had better QLQ-C30 summary scores. Two other HRQoL studies of meningioma have identified similar findings [37, 41]. It is not clear whether employment directly leads to improved HRQoL or patients remain in employment because they have good HRQoL. One study identified significantly fewer working-aged adults with meningioma were in paid employment [23]. Those that did have employment reported significantly more obstacles at work. Given that qualitative studies in other diseases have shown the benefits of employment relating to patients’ identities and self-esteem [42], future studies should evaluate methods to support patients in returning to employment and the effect of this on HRQoL. One hypothesis for this study’s findings is that meningioma patients who are employed may have better socioeconomic support and more personal resources which may confer a positive effect on their HRQoL.

Higher education level was associated with better QLQ-C30 summary scores on multivariate analysis. In another study of longer-term HRQoL outcomes in primarily surgically managed meningioma patients, lower education level was significantly associated with worse SF-36 physical component scale scores. [24] Similarly, a higher educational level was associated with better HRQoL scores in a study of patients with relapsing-remitting multiple sclerosis [43]. It is unclear how education level may impact HRQoL in meningioma patients, but patients with a higher education level may have a greater understanding and acceptance of their condition.

Multivariate analysis showed that male patients had better QLQ-C30 summary scores. A trend toward better SF-36 physical component scale scores in male patients was identified in another study of long-term HRQoL [24]. The reason for the differences between males and females is not understood but could be due to acceptance of the diagnosis and outcome.

### Study limitations and generalizability

HRQoL was not measured using a meningioma-specific patient-reported outcome measure (PROM). Therefore, there is a risk that issues of relevance to meningioma patients were not assessed. While two meningioma-specific HRQoL tools have been developed, neither was validated for use at the time of study inception [44, 45]. In the absence of a validated meningioma-specific PROM, this study utilized three validated PROMs which assessed a range of generic, cancer-specific and brain tumour-specific HRQoL issues.

Although the HRQoL domains displayed a non-normal distribution, we chose to compare the means of meningioma patients and the normative population using the independent samples t-test for this exploratory analysis. The independent samples t-test is considered stable against assumptions of non-normality. Since meningioma MCIDs are unavailable, we set the MCID level at 5 points. A previous meningioma HRQoL study utilized a higher MCID level, but since there is no evidence supporting their use for meningioma patients, we opted for lower MCIDs [23]. This study did not invite participants to respond to questionnaires as study controls, thereby limiting comparisons of meningioma HRQoL results to unadjusted normative population values. Therefore, it was not possible to control for all confounding factors such as age, sex and education level. RAND SF-36 v1 scores were compared to normative population scores assessed by the MOS SF-36 v2. The two questionnaires vary in their scoring [46]. An analysis for responder bias was not completed as the research team could only analyse data from patients who consented to participate. It is possible that patients who responded were healthier and had fewer HRQoL issues. Therefore, the HRQoL of meningioma patients may be lower than reported in this study. Finally, a small proportion of our patients had WHO Grade 2 tumours. Since these patients have a more clinically aggressive tumour, they may have inferior HRQoL scores, which may have decreased the scores of the entire cohort in some domains. A further limitation is that the multivariate analysis of HRQoL determinants was based solely on the QLQ-C30 summary score. However, this outcome was chosen as it encompasses the scores of several functioning and symptom domains. Finally, an unexpected result for QLQ-C30 financial difficulties was obtained – meningioma patients reported fewer financial difficulties than normative population values. This finding may be due to a responder bias as study participants may have a higher education level or lower deprivation index compared to the general population. Alternatively, participants may have fewer financial concerns as they may have taken early retirement due to ill health. Future studies could elucidate the long-term impact of meningioma on patients’ finances.

The results of this study are generalizable to patients who have had surgery for their meningioma or been actively monitored following a diagnosis of incidental meningioma and had more than five years of follow-up. The patients included in this study were managed at The Walton Centre NHS Foundation Trust, which is responsible for the care of patients living in both rural and urban areas. However, since this was a single-centre study from the United Kingdom, these results may not apply to patients managed in other countries and healthcare settings.

## Conclusions

This is the largest study evaluating longer-term HRQoL in patients with meningioma. The study cohort included a large group of patients with actively monitored meningioma. Longer-term HRQoL outcomes of actively monitored and operated meningioma patients were impaired. Increasing education level, employment and absence of postoperative complications were associated with better HRQoL; whereas other tumour and treatment variables were not. Our study supports the growing evidence that patients with intracranial meningioma can experience significant, longer-term consequences for their well-being, after diagnosis or treatment of meningioma. Healthcare professionals should be aware that meningioma (particularly incidental meningioma) is not simply a ‘benign’ tumour, but may have longer-term effects on health-related quality of life. An understanding of these impairments should prompt clinicians to screen for these well-being issues and appropriately offer support to assist meningioma patients as part of holistic, patient-centred care.

## Electronic supplementary material

Below is the link to the electronic supplementary material.

Supplementary Material 1

## Data Availability

Anonymized data are available (upon reasonable request) from the corresponding author.
