# Supplementary material for: Quality of life outcomes in incidental and operated meningiomas (QUALMS): a cross-sectional cohort study
Source: J Neurooncol. 2022 Dec 16;161(2):317–27. doi: 10.1007/s11060-022-04198-y (PMC9756745; doi:10.1007/s11060-022-04198-y)
Supplement: Supplementary file 1 — Supplementary Material 1 [file 11060_2022_4198_MOESM1_ESM.docx]

**Supplementary tables**

| **Domain** | **Histogram skew** | ***P* (Shapiro-Wilk test)** |
| --- | --- | --- |
| **SF-36** | | |
| Physical Functioning | Negative | <.001 |
| Emotional Well-being | Negative | <.001 |
| Social Functioning | Negative | <.001 |
| Role Physical | Negative | <.001 |
| Role Emotional | Negative | <.001 |
| Energy/ Fatigue | Negative | <.001 |
| Pain | Negative | <.001 |
| General Health | Unclear | <.001 |
| **EORTC QLQ-C30** | | |
| Physical Functioning | Negative | <.001 |
| Emotional Functioning | Negative | <.001 |
| Social Functioning | Negative | <.001 |
| Cognitive Functioning | Negative | <.001 |
| Role Functioning | Negative | <.001 |
| Fatigue | Positive | <.001 |
| Nausea & Vomiting | Positive | <.001 |
| Pain | Positive | <.001 |
| Dyspnoea | Positive | <.001 |
| Insomnia | Positive | <.001 |
| Appetite Loss | Positive | <.001 |
| Constipation | Positive | <.001 |
| Diarrhoea | Positive | <.001 |
| Financial Difficulties | Positive | <.001 |
| Global Health | Negative | <.001 |

**Supplementary Table 1.** Assessments of normality for participant SF-36 and EORTC QLQ-C30 scores.

|  | **Univariate analysis** | | **Multivariate analysis** | |
| --- | --- | --- | --- | --- |
| **Variable** | **Beta coefficient (95% CI)** | ***P* value** | **Beta coefficient (95% CI)** | ***P* value** |
| Male sex | 9.9 (2.7 to 17.1) | **.007** * | 8.8 (2.0 to 15.7) | **.012** * |
| ACCI at diagnosis | -2.4 (-4.2 to -0.7) | **.006** * | -1.2 (-3.0 to 0.6) | 0.178 ^a^ |
| Performance status | -3.8 (-8.0 to 0.4) | .073 | - | - |
| Education level | 3.5 (1.3 to 5.6) | **.002** * | 2.7 (0.5 to 4.8) | **.014** * |
| Employment | 9.5 (3.0 to 16.1) | **.005** * | 6.5 (0.1 to 12.9) | **.045** * |
| Response during/ after COVID-19 lockdown | 0.9 (-7.8 to 9.6) | .839 | - | - |
| Incidental meningioma | -1.8 (-9.4 to 5.9) | .649 | - | - |
| Skull base | 2.2 (-4.1 to 8.5) | .487 | - | - |
| Tumour laterality | 0.0 (-4.5 to 4.5) | .992 | - | - |
| Multiple meningioma | 0.0 (-10.7 to 10.7) | .997 | - | - |
| Number of AEDs | -3.6 (-9.3 to 2.2) | .224 | - | - |
| Number of surgeries | -7.0 (-15.7 to 1.6) | .110 | - | - |
| Postoperative complications | -9.1 (-15.9 to -2.3) | **.009** * | -7.9 (-14.4 to -1.4) | **.017** * |
| Number of radiotherapy courses | -2.3 (-8.3 to 3.7) | .455 |  |  |
| Type of radiotherapy | -2.1 (-5.8 to 1.5) | .246 |  |  |
| Duration of follow up | 0.3 (-0.5 to 1.1) | .473 |  |  |
| * Indicates a significant result (also underlined and in bold). ^a^ Variable excluded at step 1 of multivariate backward linear regression analysis. ^b^ Variable excluded at step 2 of multivariate backward linear regression analysis. | | | | |

**Supplementary Table 2.** Results of the univariate and multivariate linear regression analysis to identify variables significantly associated with EORTC QLQ-C30 summary score in surgically managed patients.

|  | **Univariate analysis** | |
| --- | --- | --- |
| **Variable** | **Beta coefficient (95% CI)** | ***P* value** |
| Male sex | -0.5 (-12.1 to 11.1) | .935 |
| ACCI at diagnosis | 0.1 (-2.2 to 2.4) | .929 |
| Performance status | -3.7 (-9.1 to 1.7) | .176 |
| Education level | 4.5 (0.2 to 8.7) | **.040** * |
| Employment | 8.9 (-1.3 to 19.1) | .085 |
| Response during/ after COVID-19 lockdown | -11.7 (-28.3 to 4.9) | .166 |
| Incidental meningioma | 1.6 (-16.6 to 19.8) | .859 |
| Skull base | -4.0 (-13.2 to 5.2) | .393 |
| Tumour laterality | -1.3 (-7.8 to 5.1) | .680 |
| Multiple meningioma | 13.8 (-6.2 to 33.8) | .175 |
| Number of AEDs | 18.6 (-21.0 to 58.1) | .353 |
| Intervention | 4.1 (-19.1 to 27.3) | .725 |
| Number of radiotherapy courses | 4.1 (-19.1 to 27.3) | .725 |
| Type of radiotherapy | 1.9 (-14.5 to 18.3) | .820 |
| Duration of follow up | -0.4 (-2.1 to 1.4) | .677 |
| * Indicates a significant result (also underlined and in bold). | | |

**Supplementary Table 3.** Results of the univariate linear regression analysis to identify variables significantly associated with EORTC QLQ-C30 summary score in non-surgically managed patients.
